# Increased prevalence of normal pressure hydrocephalus in both variants of frontotemporal dementia: a 10-year retrospective study

**DOI:** 10.1093/braincomms/fcad240

**Published:** 2023-09-15

**Authors:** Beatrice Pizzarotti, Gilles Allali

**Affiliations:** Neurology Service, Department of Clinical Neurosciences, University Hospital of Lausanne and University of Lausanne, 1011 Lausanne, Switzerland; Leenaards Memory Center, Department of Clinical Neurosciences, University Hospital of Lausanne and University of Lausanne, 1011 Lausanne, Switzerland

We read with great interest the article by Pouclet-Courtemanche and colleagues^1^ published recently in *Brain Communications*. The article reported an increased prevalence of idiopathic normal pressure hydrocephalus (iNPH) among patients with a behavioural variant frontotemporal dementia (bvFTD) (7.25%) in comparison with those with Alzheimer’s disease (1.1%).^1^ Therefore, we proposed to evaluate this high prevalence of iNPH in patients with FTD (not only focusing on bvFTD) followed at the Leenaards Memory Center of the Lausanne University Hospital between January 2013 and December 2022.

We selected FTD patients including bvFTD according to the Rascovsky criteria^2^ and primary progressive aphasia (PPA) according to Gorno-Tempini^3^ criteria. Our inclusion criteria were the presence of FTD without any other degenerative copathology and an available brain MRI. The diagnosis of possible or probable iNPH followed the Relkin criteria.^4^ For quantifying brain changes due to iNPH, we computed the iNPH Radscale^5^ for every comorbid iNPH patient. This study was approved by the local IRB [PACSMolis PB_2016-02582(390/15)].

We identified a total of 45 FTD patients (30 patients with bvFTD and 15 with PPA). The mean age was 72.6 years old [standard deviation (SD) 14.8], 15 females and 30 males, and the mean MoCA (Montreal Cognitive Assessment) score was 19.8/30 at the time of diagnosis. Among the 30 patients with bvFTD, the mean age was 73.4 years old (SD 14.8), 9 females and 21 males with the mean MoCA score of 18.9/30.

Among the 45 patients with FTD, 5 patients presented a comorbid iNPH (4 bvFTD and 1 PPA), corresponding to 11.1% of the FTD (13.3% for bvFTD and 6.7% for PPA). The mean Radscale was 5.6 (for the details of the subscores, see Table 1).

**Table 1:** Subject characteristics of patients with Evan’s index equal or superior to 0.3 and walking impairment

FTD variant	Age (years old)	Sex	MoCA score	Radscale total score	Evan’s index	Narrow sulci	Sylvian fissure enlargement	Focally enlarged sulci	Temporal horn	Callosal angle	Periventricular hypodensities
bvFTD	81	M	27/30	5	2	0	1	0	1	0	1
PPA	77	M	20/30	6	2	0	1	0	2	0	1
bvFTD	79	F	28/30	5	2	0	1	0	1	0	1
bvFTD	61	M	26/30	6	2	0	1	0	2	0	1
bvFTD	76	M	27/30	6	2	0	1	0	2	0	1

iNPH is frequent among older adults with a prevalence reaching around 6% in adults older than 80.^6^ Furthermore, iNPH is already known as a frequent comorbid condition in other neurodegenerative conditions, such as Alzheimer’s disease (prevalence varying from 18% to 75%, increasing with more severe cognitive deterioration^7^). Interestingly, we previously reported that gait instability could be a supportive argument for bvFTD, following the demonstration of increased gait variability in bvFTD patients in comparison with Alzheimer’s disease patients.^8^

Among the radiological features of iNPH evaluated with the iNPH Radscale, none of the patients had a narrowed vertex sulci or a sharp callosal-marginal angle that is unusual for ‘classical’ iNPH patients. In addition, the majority of the iNPH patients have an increased temporal horn that may reflect the temporal lobe atrophy found in patients with FTD. An increased temporal horn in patients with iNPH might raise the question of a degenerative comorbidity, as already suggested in a previous study.^9^

In conclusion, our 10-year study reinforces the findings of Pouclet-Courtemanche and colleagues^1^ regarding the increased prevalence of iNPH among patients with FTD. Here, we further demonstrate that this increased prevalence concerns both bvFTD and PPA variants with a 2-fold higher prevalence of iNPH in the bvFTD compared with the PPA variants.

## Data Availability

Data sharing is available upon request.
